# ALIFE@Work: a randomised controlled trial of a distance counselling lifestyle programme for weight control among an overweight working population [ISRCTN04265725]

**DOI:** 10.1186/1471-2458-6-140

**Published:** 2006-05-24

**Authors:** Marieke F van Wier, Geertje AM Ariëns, Johanna C Dekkers, Ingrid JM Hendriksen, Nico P Pronk, Tjabe Smid, Willem van Mechelen

**Affiliations:** 1 Department of Public and Occupational Health/EMGO Institute, VU University Medical Center, Van der Boechorststraat 7, 1081 BT Amsterdam, The Netherlands; 2 Body@Work, Research Center Physical Activity, Work and Health, TNO-VUmc, Van der Boechorststraat 7, 1081 BT Amsterdam, The Netherlands; 3 TNO Quality of Life, Wassenaarseweg 56, 2333 AL Leiden, The Netherlands; 4 HealthPartners, Health Behavior Group, 8100 34^th ^Avenue South, Minneapolis, MN 55440-1309, USA; 5 KLM Health Services, PO Box 7700 (SPL/AG), 1117 ZL Schiphol Airport, The Netherlands

## Abstract

**Background:**

The prevalence of overweight is increasing and its consequences will cause a major public health burden in the near future. Cost-effective interventions for weight control among the general population are therefore needed. The ALIFE@Work study is investigating a novel lifestyle intervention, aimed at the working population, with individual counselling through either phone or e-mail. This article describes the design of the study and the participant flow up to and including randomisation.

**Methods/Design:**

ALIFE@Work is a controlled trial, with randomisation to three arms: a control group, a phone based intervention group and an internet based intervention group. The intervention takes six months and is based on a cognitive behavioural approach, addressing physical activity and diet. It consists of 10 lessons with feedback from a personal counsellor, either by phone or e-mail, between each lesson. Lessons contain educational content combined with behaviour change strategies. Assignments in each lesson teach the participant to apply these strategies to every day life.

The study population consists of employees from seven Dutch companies. The most important inclusion criteria are having a body mass index (BMI) ≥ 25 kg/m^2 ^and being an employed adult.

Primary outcomes of the study are body weight and BMI, diet and physical activity. Other outcomes are: perceived health; empowerment; stage of change and self-efficacy concerning weight control, physical activity and eating habits; work performance/productivity; waist circumference, sum of skin folds, blood pressure, total blood cholesterol level and aerobic fitness. A cost-utility- and a cost-effectiveness analysis will be performed as well.

Physiological outcomes are measured at baseline and after six and 24 months. Other outcomes are measured by questionnaire at baseline and after six, 12, 18 and 24 months.

Statistical analyses for short term (six month) results are performed with multiple linear regression. Analyses for long term (two year) results are performed with multiple longitudinal regression. Analyses for cost-effectiveness and cost-utility are done at one and two years, using bootstrapping techniques.

**Discussion:**

ALIFE@Work will make a substantial contribution to the development of cost-effective weight control- and lifestyle interventions that are applicable to and attractive for the large population at risk.

## Background

As is the global trend, more than 5.6 million adults in the Netherlands (i.e., 46.1% of the population of 20 years and older) are overweight and numbers are still rising [1]. Overweight is defined as having a body mass index (BMI) of 25 or higher. It is a risk factor for a range of health problems, most notably those related to the metabolic syndrome. This syndrome is characterised by abdominal adiposity and several associated metabolic anomalies like insulin resistance, dyslipidaemia and hypertension. These metabolic changes are related to the development of coronary heart disease and type 2 diabetes mellitus. The growing prevalence of overweight and the increase in severity will result in an increase in these diseases. We have to look at ways to turn this tide.

Research shows that intentional weight loss in overweight people with symptoms of the metabolic syndrome, produces a significant improvement in these symptoms and reduces the risk for developing diabetes [2-4]. Reduction in overall mortality as a result of intentional weight loss still needs to be established [5-7], but research shows that maintaining a normal BMI during adulthood protects against diabetes and cardiovascular disease [4]. Therefore, preventing weight gain is crucial in both people who are already overweight, as well as in those who are at risk for becoming overweight.

Overweight develops when the energy balance between physical activity and foods eaten, is generally positive [8]. Prevention of weight gain should concentrate on restoring this balance by increasing physical activity and improving quality of diet. In those seeking weight loss, a negative energy balance should be achieved. Both an increase in physical activity and a well balanced diet have positive effects by themselves. Physical activity reduces blood pressure, improves lipid profiles and protects against cardiovascular disease, type 2 diabetes and certain types of cancer [9]. A diet rich in fruit, vegetables and unsaturated fatty acids helps to lower blood pressure and blood total cholesterol, and prevents against the development of cardiovascular diseases [10]. Improvement of physical activity and diet therefore not only has positive effects on weight control, but also contributes to the amelioration of risk factors for coronary heart disease and type 2 diabetes mellitus. Weight control (i.e., weight loss, maintenance of lost weight and prevention of weight gain) interventions should promote these lifestyle changes.

While there is consensus on the incorporation of both diet and physical activity for weight control, less is known about how these behaviours can be sustainably changed. Most research in the field of weight control has concerned weight loss and to a lesser extent maintenance of lost weight. Little research has been directed towards prevention of weight gain and effective interventions still need to be determined [11]. Behavioural and cognitive behavioural therapies are commonly used for weight loss interventions [12]. They facilitate better maintenance of weight loss than other interventions. These therapies can be used either in a group approach as well as in individual treatment. In both approaches, multiple visits to a treatment centre are required. This is demanding for both the patient as well as the health care system and makes this mode of delivery not well suited for the large section of the population that needs to be reached. New approaches are therefore necessary. Two studies in the United States show that distance counselling programmes (i.e., personal coaching by mail, phone or e-mail) based on behavioural principles can be successful in producing weight loss [13,14]. Distance counselling promises to be cheaper and more accessible than traditional treatments. However, effects on weight maintenance and prevention of weight gain, changes in diet and physical activity, changes in metabolic risk factors and cost effectiveness still have to be established. The ALIFE@Work trial is designed to study these outcomes. This trial is carried out in a working population. Increasing numbers of employees have a sedentary profession and are therefore at risk of developing overweight, or are already overweight. Research shows that productivity losses due to overweight can amount to 10% [8]. Offering distance counselling to the working population might be a feasible and cost-effective way to reach a large group of those in need to change their lifestyle.

The main objective of the ALIFE@Work study is to evaluate, among an overweight working population, the effectiveness of a lifestyle intervention programme on body weight, physical activity and eating habits. Secondary objectives of this study are 1) to compare the effectiveness of the use of two different communication technologies, i.e., telephone and internet, and 2) to evaluate the cost-effectiveness of this lifestyle programme. In this article we describe the design of the study and the participant flow up to, and including randomisation.

## Methods/Design

ALIFE@Work is a randomised controlled trial with a two-year follow-up. Randomisation takes place to three groups: to a control group (control) and to an intervention programme either counselled by phone (phone), or counselled by email (internet). The recruitment and data collection for this study started in January 2004. Data collection will continue until September 2006.

The study design, procedures and informed consent form were approved by the Medical Ethics Committee of the VU University Medical Center (under number 03/193), and all participants provided written informed consent.

### Study population

Participants are employees of seven different companies in the Netherlands, including two IT-companies, two hospitals, an insurance company, the head office of a bank and a police force. Of the approximately 21,000 employees working at these companies about 40%, i.e. 8,400, were estimated to be overweight. All 21,000 employees were approached per company, through an invitational letter and a screening questionnaire. This was done in six months time, with an interval of three weeks to one month between the companies. The screening questionnaire included demographic questions and questions on body weight and body height.

### Inclusion & exclusion criteria

Inclusion criteria of the study were: 1) BMI ≥ 25 kg/m^2^, 2) paid employment of at least 8 hours a week, 3) adequate knowledge of the Dutch language, 4) access to internet and skilled in using it, 5) at least 18 years of age. Employees were excluded for the following reasons: pregnancy, diagnosis or treatment of cancer, any other disorder that makes physical activity impossible.

### Sample size

A power calculation was carried out for the main outcome variable, i.e. weight change. Calculations were made for a comparison between two equal size groups. Power calculation for weight change was carried out for three levels of power, namely 80%, 85% and 90%. The standard deviation (SD) for two-year weight loss was expected to be 6.8 kg, based on prior weight treatment studies [15,16]. Calculations showed that differences in mean two-year weight loss of about 1.4 kg between conditions will be detectable with 90% power in two-tailed tests with a significance level of 0.05 for a sample of 500 employees in each group. The sample size for the study was therefore determined at 1500.

### Randomisation

Randomisation took place at an individual level. After baseline measurements, the employee was randomised to one of the three study groups and either to a group receiving basic weight measurements (80% of each study group) or to a group receiving additional measurements (20% of each study group). This two-step randomisation meant that there were six groups an employee could be assigned to. Randomisation to these six groups was done by block randomisation, with each block containing 15 allocations. A computerized random number generator drew up an allocation schedule. An administrative assistant put the group allocation in opaque sealed envelopes, numbered 1 to 1,500. These envelopes were taken to the locations of the baseline measurements and opened in the given order. The researchers were blinded for the allocation schedule, but were not blinded for allocation after randomisation. The participants were, in consequence of the nature of the intervention, not blinded for allocation after randomisation. Employees were not allowed to change groups after randomisation.

### Study groups

#### Control group

The employees in the control group received three information brochures with general information on overweight, physical activity and nutrition, and a calorie chart. These materials were briefly explained to the employee. All materials were published by the Netherlands Heart Foundation, for general use.

#### Intervention groups

Both intervention groups received a lifestyle intervention programme. This intervention was similar in content, but differed in the way the content was distributed to them and in the way the participant communicated with a personal counsellor that was appointed to them. The intervention conditions are described later on.

### Participant flow

**Figure 1:**
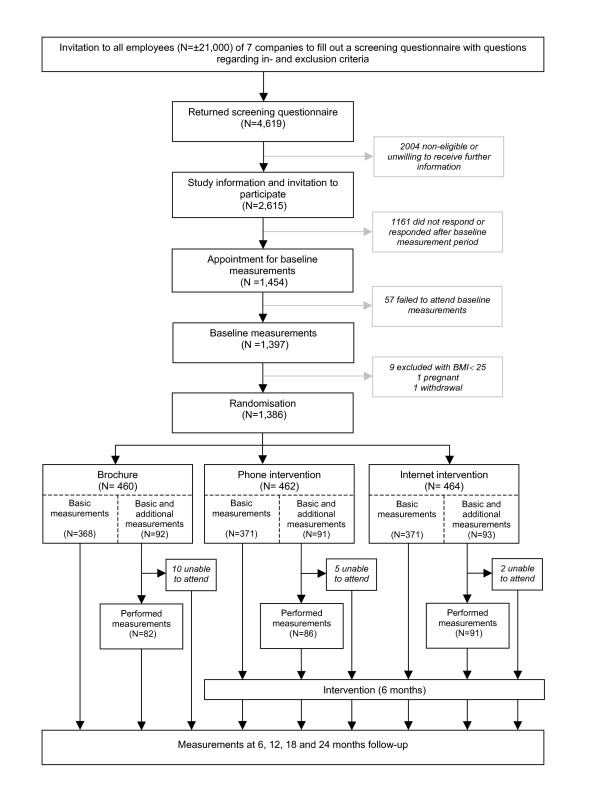


The study design and participant flow (achieved at recruitment closure and brought up to closure of the baseline measurement period, August 2004) are shown in Figure 1.

Out of a total of approximately 21,000 employees, 4,619 returned the screening questionnaire. Of these, 2,615 were eligible and had no objections to receiving further information. They received an information brochure in which the study protocol was clearly described. Employees were free to further inquire about the study without any engagement. 1,454 employees were willing to participate and applied in time. They were all individually invited for a first appointment in which the basic measurements body weight and body height were assessed by the researchers. From these measurements, BMI was calculated. Those with a BMI lower than 25 kg/m^2^, which was the case for nine employees, were excluded. One employee became pregnant between screening and baseline measurements and was also excluded. One employee withdrew before randomisation. 57 employees did not show up for the baseline measurements. Therefore, 1,386 employees were randomised to the two intervention conditions (phone group N = 462, internet group N = 464), or to the control group (N = 460). Within these groups a number of subjects received additional measurements as follows: phone group N = 91, internet group N = 93 and control group N = 92. A second appointment was arranged within one week after randomisation, to perform these measurements. In each group a few employees were unable to attend the additional measurements: five out of the phone group, two out of the internet group, and ten out of the control group.

### Intervention

#### Intervention programme

The lifestyle intervention programme is an adapted version of previous work of HealthPartners in Minnesota, USA [13]. This Dutch version of the lifestyle programme is called "Leef je Fit". The programme is based on social cognitive theory [17] and emphasizes 1) identifying behaviours in need of change, 2) setting goals for change, 3) monitoring progress, 4) modifying environmental cues to facilitate change, and 5) modifying consequences to motivate change. An essential part of the programme is coaching on these activities by a personal counsellor.

The programme consists of ten lessons or 'modules'. These provide information on nutrition and physical activity, and teach techniques for changing behaviour (e.g., self-monitoring). Assignments in the modules assist in applying these techniques. The programme can be worked through either at work or at home.

Participants are asked to study a module and finish the assignments every two weeks, so they are able to finish the programme within six months. After finishing each module, participants are contacted by their counsellor. Counselling is done according to two comparable standardized counselling protocols (i.e., for the two communication methods).

Next to the programme materials, the participant receives a pedometer at the start of the programme.

#### Phone intervention

Programme materials for the phone group are provided after randomisation. The employee receives a binder with the ten modules, and the same brochures that are given to the control group. The intervention starts when the personal counsellor first calls the participant, about two weeks after randomisation. In this first contact the counsellor explains the workings of the programme and sets a time and date for the next contact in which the first module will be addressed. In between these contacts, the employee studies the module and fills out the assignments. The counsellor calls the employee at the scheduled date and time, to go through the module and to talk about the assignments according to the standardized protocol. At the end of the call, a new date and time are set for the next call about two weeks later. This interactive process continues until the employee completes all modules, or until the participant declines contact. Participants also have the possibility to contact their counsellor by phone between modules, if they have additional questions.

#### Internet intervention

A website is developed for the intervention. The internet-based lifestyle intervention starts when the employee receives a welcome e-mail, about two weeks after randomisation, with a unique username and password. The first time an employee logs on to the site, he is directed to an introduction which explains the workings of the programme. The employee is asked to fill out some personal details for the counsellor. The employee then starts with the first module. The assignments are filled out on the site and stored in a personalized area of the website. The personal counsellor receives an alert when the employee has finished a module. The counsellor then checks the assignments and comments on them through e-mail within 5 working days, according to the standardized protocol. One week after finishing a module, the participant is able to start with the assignments of the next module. Modules have to be worked through in the given order.

When an employee does not log on to the website according to schedule, he receives an e-mail reminder twice a week. These reminders are continued for the full duration (i.e., six months) of the programme. Besides being reminded by e-mail, employees can also choose to be reminded by text message on their mobile phone.

Participants have the possibility to e-mail their counsellor between modules, if they have additional questions.

#### Counselling

Four counsellors with a degree in nutrition or physical activity provide coaching for the participating employees. Prior to the start of the intervention, they receive a four week training. The counsellors are made familiar with the principles behind the programme and go through the intervention materials. A psychologist trains them in counselling techniques. A pilot group is put together to give the counsellors the opportunity to practise with the counselling protocols, counselling skills and administrative procedures.

Counselling takes place according to a standardized protocol, which is developed for each communication method. Both protocols are similar to each other in approach.

The protocol for the phone-condition consists of a timed outline for each module and provides the counsellors with guidelines on how to counsel the assignments.

The protocol for the internet-condition consists of a semi-prepared e-mail for each module. The e-mail discusses the module by giving generic comments. To this the counsellor adds the answers the participant has filled out in the assignments and addresses those individually in the same manner as in the phone calls.

In order to keep track of appointments, purport of phone calls and e-mails, and the progress of the participants, a web-based participant management system is developed. Counsellors have access to the assignments and progress from the internet group through this participant management system.

### Outcome measures, confounding- and mediating variables

Primary outcomes of the study are 1) weight change and change in BMI, 2) change in physical activity level, and 3) change in dietary intake of fat, fruit, vegetables, sugar and alcoholic beverages. Effects in both intervention groups combined against the effects in the control group are studied, as well as effects in the phone group against effects in the internet group.

Besides these primary outcomes, other outcomes are studied also. Among those are changes in waist circumference, sum of skin folds, blood pressure, total blood cholesterol and aerobic fitness. Other outcomes that are studied include change in work performance/productivity, perceived health, empowerment, stage of change and self efficacy concerning weight control, physical activity and eating habits. Changes in direct medical costs and changes in indirect costs for sickness absence and loss of work productivity are studied as well.

In conclusion, a process evaluation of the execution of the intervention and of participant satisfaction is carried out after completion or the intervention.

Assessment of the aforementioned outcomes, is done either by questionnaire or by physiological measurements. Physiological measurements take place at the work site or near the work site. Questionnaires are sent to the home address of the participant. The scheduling of all measurements is shown in Table 1.

#### Primary outcome measures

##### Body weight and BMI

Body weight and body height are assessed in all participants. Body weight is measured in kg, to the nearest 0.1 kg, with a digital scale (Seca 770; Seca GmbH & Co, Hamburg, Germany). Participants are wearing light clothing and no shoes. Body height is measured in m, to the nearest 0.001 m, with a portable stadiometer (Seca 214, Leicester Height Measure; Seca GmbH & Co, Hamburg, Germany). Positioning of the body is standardized by asking the subject to stand straight, without shoes and with the heels together. Both weight and height are measured twice, and for each mean value of the two measurements is computed. BMI is calculated by dividing the measured body weight (kg) by the squared measured body height (m).

In addition, in a questionnaire self-reported body weight is assessed. Participants are asked to weigh themselves wearing light clothing and no shoes.

##### Dietary intake and physical activity

Dietary intake and physical activity are assessed by means of a questionnaire. The focus of dietary intake is on fat, fruit and vegetable intake. Fat intake is assessed by the validated Dutch Fat List [18]. Fruit and vegetable intake are asked with a short fruit and vegetable questionnaire, that has been validated as well [19,20]. Intake of sugar and alcoholic beverages is assessed with a set of questions which were developed for this study. Physical activity level is assessed with the validated Short Questionnaire to Asses Health enhancing physical activity (SQUASH) [21].

#### Physiological outcome measures

For a random sample of each group, several additional physiological measurements are assessed in the following order: waist circumference, sum of skin folds, blood pressure, total blood cholesterol, and aerobic fitness. They are all done according to standardized protocols and take approximately 45 minutes per session.

##### Waist circumference

Waist circumference (in cm) is measured twice with a measuring tape (Gulick; Creative Health Products, Ann Arbor, MI, USA) with a range of 0–150 cm. Waist circumference is measured to the nearest 0.1 cm, at the midpoint between the lower border of the ribs and the upper border of the pelvis. A mean value of the two measurements is computed.

In addition to the objectively assessed waist circumference in the sub-sample, all 1386 employees are asked to report their self measured waist circumference in each questionnaire. For that aim, a measuring tape is sent to all participants along with the baseline questionnaire. This measuring tape is developed for the study and has a range of 0–135 cm. Participants get instructions on how to use the measuring tape and are asked to report their waist circumference to the nearest cm.

##### Sum of skin folds

According to the method of Weiner and Lourie [22] the following four skin folds are measured twice with a Harpenden caliper (HSK-BI; Baty International, Burgess Hill, UK) up to the nearest 0.1 mm and on the right side of the body: sub scapular, suprailiac, triceps and biceps. In case the two measurements of a fold differ more than 1.0 mm, the skin fold is measured a third time. Next, for each skin fold, a mean value of the measurements is computed and the four skin folds are added up.

##### Blood pressure

Blood pressure is measured with a fully automated blood pressure monitor (Omron HEM 757E [M5-I]; Omron Healthcare Europe BV, Hoofddorp, The Netherlands). This blood pressure monitor is validated and recommended for clinical use [23]. A regular size cuff is used on the right upper arm. When the subject has an upper arm circumference of 33 cm or more, a large size cuff is used. The right arm is placed on a table so as the cuff is on a level with the heart. After the employee has rested for 5 minutes in a sitting position, blood pressure is measured twice. A mean value of the two measurements is computed.

Employees that have a blood pressure of 140/90 mmHg or higher are advised to visit their general practitioner.

##### Total blood cholesterol

Total blood cholesterol level is determined with the Reflotron^® ^Plus (Roche Diagnostics GmbH, Mannheim, Germany) portable blood analysis system. The Reflotron^® ^Plus provides a good risk classification [24]. Total cholesterol is determined in non-fasting capillary blood, collected from a finger prick. If total cholesterol is lower than 3.0 mmol/l or exceeds 6.5 mmol/l, the measurement is repeated. If both measurements are over 6.5 mmol/l, the employee is advised to visit his general practitioner.

##### Aerobic fitness

Aerobic fitness is assessed by means of the Chester Step Test (CST) [25]. The CST is a sub-maximal test that gives a reliable prediction of VO_2_max [26]. The employee is asked not to drink coffee or smoke two hours prior to the test. During the CST, the employee steps on and off a 10, 15, 20 or 25 cm gym bench. The height of the bench depends on the participant's age and current fitness level, as described in the manual of the test [27]. The test starts at the pace of 15 steps per minute. The pace increases every two minutes to 20, 25, 30 and 35 steps per minute, respectively. A metronome sets this stepping rate. The heart rate of the participant is monitored continuously by means of a heart rate monitor (Polar S610; Polar Electro Oy, Kempele, Finland). Also, the subject must report his subjective rate of exertion at the end of each stage, using a Borg scale ranging from 6 tot 20 [28]. The test is terminated at the end of a stage at which the subject's heart rate reaches 80% of his age predicted maximal heart rate (220 minus age), or when the reported rate of perceived exertion exceeds 14 (hard) [27]. The estimated VO2max is calculated with calculator software that comes with the Chester step test (ASSIST creative resources Limited, Redwither Business Park, UK).

#### Other outcome measures

Perceived health and empowerment are respectively assessed with the RAND-36[29] and the Mastery Scale of Pearlin [30]. Stage of change in relation to weight control, physical activity and eating habits is assessed using questions about intentions to start changing these behaviours [31-33]. The behavioural determinant self efficacy, concerning weight control, physical activity and eating habits is assessed with a set of questions developed for this study.

The cost-effectiveness and the cost-utility of the lifestyle programme will be ascertained. The cost-effectiveness analysis will be performed from both a company perspective as well as from a societal perspective. The primary outcome measures weight change, change in physical activity level, and change in dietary intake will be included in this cost-effectiveness analysis.

For the company perspective, intervention costs will be compared with obtained benefits from reduced sickness absence and increased work productivity. Intervention costs include costs for the development of the intervention, such as the development of the website and the binders, and costs for the implementation of the intervention, such as salary costs of the counsellors and costs for hosting the website. Self-reported sickness absence is prospectively measured by keeping a six-monthly diary. On a monthly basis the employee records full days absence from work due to sick leave. Diaries are provided at the start of each six month measuring period. In addition, data on sickness absenteeism will be derived from employer records, from six months before baseline till two years after baseline. Work productivity is measured with the work related questions from the WHO Health and Work Performance Questionnaire (HPQ) [34]. Work productivity assessed by the HPQ is based on self-report. The HPQ is validated and available in several languages, including Dutch.

The cost-effectiveness from a societal perspective will be addressed by assessing health care utilization and medical costs. Health care utilization is based on actual resource use, using prospective data collected by a diary in which the employee records use of medical services and medication on a monthly basis. This diary is combined with the diary recordings on sickness absence. Direct medical costs of health care utilization are calculated using cost prices if available and otherwise tariffs will be used.

Utilities for the cost-utility analysis are based on the EuroQol [35].

#### Confounding and mediating variables

Possible confounders that are assessed include certain demographics, smoking behaviour, health conditions, weight outcome evaluation and weight control behaviours. Demographic variables that are measured are age, educational level, personal income, country of birth, gender, marital status and number of adults and children living in the employee's household. Smoking is defined as use (yes/no) of cigarettes, or other tobacco products (cigars, pipes). Having quit smoking (yes/no) in the past six months is also assessed. Health conditions related to overweight, physical inactivity and unhealthy eating habits are assessed using a series of questions about the use of medication for hypercholesterolemia, hypertension, angina, heart disease, myocardial infarct, depression and diabetes. Weight outcome evaluation is assessed by three questions concerning acceptable weight, desirable weight in six months and the wish for weight loss or weight management. Weight control behaviours are measured by reports of frequency of self weighing, frequency of weight loss attempts in the last two years, and employed methods for weight control.

#### Process evaluation

The process evaluation among participants assesses the participants opinion of the allocated study group, use and appraisal of the components of the lifestyle intervention programme or (in the case of the control group) of the provided general brochures, evaluation of coaching by the personal counsellor (if applicable) and a generic grade for the programme. Additional questions are asked about contentment with achieved results, reasons for not finishing the programme, and how much the participant would be willing to pay for the offered programme.

Objective measures for use of both intervention methods will be obtained from the database that underlies the participant management system and the intervention website.

### Statistical analyses

Baseline values will be analysed for differences in the three groups, by one-way-analysis of variance for numerical data and chi-square for categorical data.

Analyses for the primary outcomes, for the physiological outcomes and for the other outcomes (e.g., perceived health) will be at six months (short term) and after two years (long term). They will be performed according to the intention-to-treat principle. Analyses for short term results will be performed with multiple linear regression. Analyses for long term results will be performed with multiple longitudinal regression.

Bootstrapping will be used for comparison of mean direct, indirect and total costs between groups [36]. Confidence intervals for the mean difference in costs will be obtained by bias corrected and accelerated bootstrapping. Cost-effectiveness ratios and cost-utility ratios will also be calculated with bootstrapping according to the bias corrected percentile method. Separate cost-effectiveness ratios will be calculated for the primary outcomes [37]. Bootstrapped cost-effect pairs will be graphically represented on a cost-effectiveness plane [38]. Acceptability curves will be calculated, which show the probability that a treatment is cost-effective at a specific ratio.

## Discussion

The ALIFE@Work study is motivated by the increasing prevalence of overweight and obesity [1]. Overweight is a risk factor for, among others, cardiovascular diseases and type 2 diabetes [5]. The study aims to result in sustainable changes in physical activity and diet, to enhance weight control. Thus, by inducing weight control, ALIFE@Work aims to limit the future burden of overweight and obesity related diseases.

The rising prevalence of overweight and obesity has created a need for cost-effective lifestyle interventions that can reach a broad population on an individual level. One approach is behaviour counselling based on social cognitive theory. This is shown to be effective for weight loss, but has not yet been applied to the prevention of weight gain [11,12]. Counselling programmes usually require participants to visit a treatment centre during office hours and to do so on a regular basis. This is both demanding for the health care system as well as for the participant. Distance counselling channels like phone and internet could be feasible ways to offer the same counselling in a more cost-effective way. Two other studies have looked at the effect of counselling by either phone or e-mail on weight loss [13,14]. ALIFE@Work is the first study to allow for a direct comparison of the effects of these different types of distance counselling. Also, it is the first study to apply personal counselling to the prevention of weight gain.

The target group of the intervention are employees that are approached through their employer. Working people represent a large part of the population at risk, especially since most jobs have become sedentary. Also, approaching people through their employer is a new way to reach individuals that might otherwise not partake in weight control programmes. The approach is similar to the way the programme could be implemented, if proven cost-effective.

Because the future effects of the intervention on the prevalence of overweight related diseases and mortality are hard to study, intermediate endpoints are chosen. Short-term effects (6 months) and long term effects (24 months) on weight and BMI, physical activity and eating behaviour are studied. A strong point of the ALIFE@Work study is the follow-up of two years, which makes it possible to evaluate weight loss and weight management up till one-and-a half years after the intervention is finished.

Overweight is associated with (abdominal) adiposity and other cardiovascular risk factors, such as hypercholesterolemia and hypertension. As the main outcomes weight and BMI are associated with, but not directly reflect (abdominal) adiposity, waist circumference and sum of skin folds are assessed in a sub-sample of the study population. Aerobic fitness is assessed in the same sub-sample to serve as a proxy for physical activity, which is otherwise measured by self report. Blood pressure and total blood cholesterol are also studied in the sub-sample. These physiological measures add outcomes that are one step further in the chain of the development of cardiovascular disease. Also, ALIFE@Work is one of the few lifestyle intervention studies that evaluates the effect on biological cardiovascular risk factors in overweight adults who are apparently healthy [5].

Cost-effectiveness and cost-utility for both counselling strategies will be assessed at one- and at two-years follow-up. As far as we know, economic evaluations from the societal perspective of weight control interventions based on change in physical activity, diet or behaviour counselling have not been performed as yet [39,40]. Our study would be the first to give information based on real costs and outcomes.

In conclusion, principal findings of the ALIFE@Work study concern the (cost-) effectiveness of a 6-month lifestyle intervention based on social cognitive theory and personal counselling by phone or e-mail, in an overweight but otherwise healthy working population. Due to report in 2007, the ALIFE@Work study has the potential to make a substantial contribution to the development of cost-effective weight control- and lifestyle interventions that are applicable to and attractive for the large population at risk.

## Competing interests

The author(s) declare that they have no competing interests.

## Authors' contributions

WM is the Principal Investigator, who conceived of the idea, obtained funding for the study, helped to recruit companies and advised on the conduct of the study. GA is co-applicant of the grant and provided advice and guidance on the study design and the conduct of the study till June 2005. IH and NP advised on the study design. TS helped to recruit companies and advised on the study design. MW and CD developed the interventions and are the study investigators.

All authors read and approved the final manuscript.

## Pre-publication history

The pre-publication history for this paper can be accessed here:



## Figures and Tables

**Table 1:** Schedule of measurements

**Outcome measures**	D, Q, O*	**Baseline**	**6 months**	**12 months**	**18 months**	**24 months**
*Body weight*	D					
	Q					
*Body height*	D					
	Q					
*Dietary intake*	Q					
*Physical activity*	Q					
*Waist circumference*	D					
	Q					
*Sum of skin folds*	D					
*Blood pressure*	D					
*Total blood cholesterol*	D					
*Aerobic fitness*	D					
*Perceived health*	Q					
*Empowerment*	Q					
*Stage of change*	Q					
*Self efficacy*	Q					
*Work performance*	Q					
*Confounding and mediating variables*	Q					
*Sickness absence and health care utilization*	O^1^					
*Programme costs*	O^2^					
*Data for process evaluation*	Q					
*Data for process evaluation*	O^3^					

Outcome measures are directly measured (D), measured by questionnaire (Q) or measured otherwise (O);

^1 ^Sickness absence data and data on health care utilization are collected by a six-monthly diary. Sickness absence data are also derived from employer payroll records, from six months before baseline till two years after baseline;

^2 ^Programme costs are assessed continuously during development and implementation of the programme;

^3 ^Participants appraisal of the programme is measured by questionnaire at six months after baseline. Objective measures are collected from the database underlying the participant tracking system and the website.

continuous measurement from this period till next period
